# Genetic Testing Strategies in Newly Diagnosed Endometrial Cancer Patients Aimed at Reducing Morbidity or Mortality from Lynch Syndrome in the Index Case or Her Relatives

**DOI:** 10.1371/currents.eogt.b59a6e84f27c536e50db4e46aa26309c

**Published:** 2013-09-16

**Authors:** Alison Stewart

**Affiliations:** (1) McKing Consulting Corp., and (2) Centers for Disease Control and Prevention

## Abstract

Endometrial cancer is the first malignancy in 50% of women with Lynch syndrome, an autosomal dominant cancer-prone syndrome caused by germline mutations in genes encoding components of the DNA mismatch repair (MMR) pathway. These women (2-4% of all those with endometrial cancer) are at risk of metachronous colorectal cancer and other Lynch syndrome-associated cancers, and their first-degree relatives are at 50% risk of Lynch syndrome. Testing all women newly diagnosed with endometrial cancer for Lynch syndrome may have clinical utility for the index case and her relatives by alerting them to the benefits of surveillance and preventive options, primarily for colorectal cancer. The strategy involves offering germline DNA mutation testing to those whose tumour shows loss-of-function of MMR protein(s) when analysed for microsatellite instability (MSI) and/or by immunohistochemisty (IHC). In endometrial tumours from unselected patients, MSI and IHC have a sensitivity of 80-100% and specificity of 60-80% for detecting a mutation in an MMR gene, though the number of suitable studies for determining clinical validity is small. The clinical validity of strategies to exclude those with false-positive tumour test results due to somatic hypermethylation of the MLH1 gene promoter has not been determined. Options include direct methylation testing, and excluding those over the age of 60 who have no concerning family history or clinical features. The clinical utility of Lynch syndrome testing for the index case depends on her age and the MMR gene mutated: the net benefit is lower for those diagnosed at older ages and with less-penetrant MSH6 mutations. To date, women with these features are the majority of those diagnosed through screening unselected endometrial cancer patients but the number of studies is small. Similarly, clinical utility to relatives of the index case is higher if the family’s mutation is in MLH1 or MSH2 than for MSH6 or PMS2. Gaps in current evidence include a need for large, prospective studies on unselected endometrial cancer patients, and for health-economic analysis based on appropriate assumptions.

## **Clinical scenario

Approximately 2-4% of endometrial cancer (10% in women diagnosed under the age of 50) is attributable to Lynch syndrome,^1^^,^ 2 ^,^ 3 ^,^ 4 an autosomal dominant cancer-prone syndrome caused by germline mutations in the *MLH1*, *MSH2*, *MSH6 *or *PMS2 *genes, which encode components of the DNA mismatch repair (MMR) pathway, or, in a small proportion of cases, by deletions in the *EPCAM *gene that lead to epigenetic silencing of the adjacent *MSH2 *gene (reviewed in 5). Individuals with Lynch syndrome are at increased risk for cancers of the colon, rectum, endometrium, ovary, small bowel, urothelium, pancreas, biliary tract, stomach, brain, skin and possibly breast (reviewed in 6). In 50-60% of women with Lynch syndrome, endometrial cancer is the first malignancy.^7^^,^ 8 Those who have already been diagnosed with cancer are also at risk of developing a Lynch-syndrome-associated cancer at another site, or a second primary cancer in the same organ.

If Lynch syndrome is suspected in a cancer patient, DNA testing can be used to determine whether the patient has a MMR or *EPCAM *gene mutation. However, because the prevalence of Lynch syndrome amongst those diagnosed with cancer is low, even for the two most common Lynch Syndrome-associated cancers (colorectal and endometrial), germline DNA testing for all cancer patients is not currently feasible so various clinical triage approaches have been developed to identify the subset of patients most likely to have Lynch syndrome. Triage has typically been based on factors such as family history of Lynch syndrome or Lynch-syndrome-associated cancers, age at diagnosis, and features of the primary tumour (usually assumed to be colorectal).^9^^,^ 10 However, such criteria have been criticised as lacking adequate clinical validity for identifying endometrial cancer patients who should be offered genetic testing.^2^^,^ 11 ^,^ 12 ^,^ 13 ^,^ 14 ^,^ 15 Inadequate clinical validity in unselected endometrial cancer patients with Lynch syndrome has also been found for a variety of clinical prediction rules (PREMM_1,2,6_, MMRpredict and MMRpro) developed to predict the probability of a Lynch syndrome mutation in cancer patients.^16^^,^ 17 ^,^ 18 ^,^ 19 Young age at diagnosis (usually <50 years) has also been criticised as likely to miss 30-70% of endometrial cancer patients with Lynch syndrome.^11^^,^ 13 ^,^ 14 ^,^ 20

To improve the sensitivity of detecting Lynch syndrome among endometrial cancer patients, tumour testing by microsatellite instability (MSI) and/or immunohistochemistry (IHC) analysis has been suggested for all newly diagnosed patients.^2^ If this analysis indicates that one or more of the MMR proteins is absent or non-functional in the tumour, the patient would be referred for further investigation and, if sporadic cancer can be excluded, offered germline DNA testing for MMR mutation(s). Those who test positive for a Lynch syndrome mutation can enter surveillance programmes or be offered preventive interventions with the aim of reducing mortality and morbidity from metachronous Lynch syndrome cancers. In addition, their relatives can be offered diagnostic testing for Lynch syndrome, with subsequent risk-reducing surveillance or prevention for those who test positive.^2^^,^ 13 An analogous strategy has been found to have clinical utility for the relatives of newly diagnosed colorectal cancer patients and has been recommended by the Evaluation of Genomic Applications in Practice and Prevention (EGAPP) Working Group.^21^^,^ 22 ^,^ 23 ^,^ 24

In some centres in the US, screening of all newly diagnosed colorectal cancer and/or endometrial cancer patients by tumour testing is already being carried out. A recent survey identified 29 insitutions screening all colorectal tumours, and 11 screening all endometrial tumours.^25^

The aim of this paper is to gather and present evidence on offering molecular tumour testing for Lynch syndrome to all newly diagnosed endometrial cancer patients, with the aim of reducing morbidity or mortality from subsequent cancers in the index case, and/or from Lynch syndrome cancers in her relatives.

## Test description

Loss or malfunction of the MMR system leads to numerous unrepaired errors in the genome. Some of these errors are manifested as changes in the lengths of short repeated sequences known as microsatellites. Testing for microsatellite instability (MSI) is done by comparing the distribution of PCR-amplified microsatellite fragment lengths between tumour and normal tissue.^26^ Tumours in which more than 30% of the microsatellites are unstable are classified as high-frequency instability (MSI-H). A low frequency of instability (MSL-L) is one in which one or up to 30% of the markers show instability, while a microsatellite-stable (MSS) tumour shows no unstable markers.^5^^,^ 26 In 1998 a panel of 5 microsatellite markers, including 2 mononucleotide markers, was recommended in the proceedings of a workshop convened by the National Cancer Institute (NCI), at which the Bethesda guidelines were established.^27^ More recently, incorporation of additional mononucleotide markers has been found to improve sensitivity.^21^

In the IHC method, loss of MMR expression is determined by lack of immunohistochemical staining of MMR proteins (MLH1 MSH2, MSH6, PMS2) in the nuclei of tumour cells.^5^ Because it detects the absence of specific MMR proteins, IHC can also inform subsequent genetic testing for germline mutations. IHC is performed on slices of paraffin-embedded tumour tissue transferred to microscope slides. Nuclei showing any detectable staining (>1%) are scored as positive. The MMR proteins function as heterodimeric complexes: MLH1 with PMS2 (or PMS1) and MSH2 with MSH6 (or MSH3).^28^ As some of these proteins are unstable when not paired in a complex, a defective MMR system may involve loss of expression of more than one protein: tumours of individuals with germline *MLH1 *mutations generally lack both MLH1 and PMS2 expression, while those with a germline *MSH2 *mutation (or, more rarely, a deletion in the *EPCAM *gene) lack both MSH2 and MSH6 expression. However, germline mutations in *MSH6 *or *PMS2 *do not result in loss of MSH2 or MLH1; therefore, tumours with isolated loss of MSH6 or PMS2 expression indicate a possible germline mutation in the respective gene.^29^

Tumour testing by MSI and/or IHC typically yields abnormal results in 15-25% of unselected endometrial cancer patients.^4^^,^ 19 As the current cost of germline DNA testing for such a large number of patients would be prohibitively high in a population screening programme, further tumour analysis may be undertaken to identify those tumours that are likely to be sporadic.^30^ Most of these are tumours that show an MSI-H phenotype and loss of MLH1 and PMS2 expression as a result of somatic methylation of the *MLH1*promoter.^31^ In colorectal cancer, sporadic cancers can be distinguished from Lynch syndrome cancers by testing for the V600E mutation in the *BRAF *gene, which is frequently mutated in the former but not the latter, and is strongly associated with *MLH1 *promoter methylation.^32^^,^ 33 In contrast, the frequency of *BRAF *gene mutations in endometrial tumours is thought to be low (no higher than 1%), regardless of methylation status, suggesting that *BRAF *gene analysis is not useful in testing of endometrial cancer patients.^4^^,^ 34 ^,^ 35 If MLH1 expression is absent in endometrial tumours, the methylation status of the *MLH1 *promoter can be tested directly, for example by bisulphite treatment of the DNA (which converts all unmethylated cytosines to uracil) followed by sequencing or restriction analysis or methylation-specific PCR (MSP).^1^^,^ 2 An alternative method is methylation-specific multiplex ligation-dependent probe amplification (MS-MLPA).^4^

Germline DNA testing in peripheral blood or normal endometrium is usually carried out by direct gene sequencing of the *MLH1*, *MSH2*, *MSH6 *and (in some laboratories) *PMS2 *genes, together with a method such as MLPA to detect large genomic rearrangements.^2^^,^ 4 MLPA can also be used to detect deletions in the *EPCAM *gene.^36^ MMR gene mutations are classified as deleterious if they are predicted to encode a truncated or unstable protein (e.g. frameshift, nonsense, splice site). Missense mutations may often have uncertain clinical significance.

In some patients with tumour test results suggestive of Lynch syndrome, no mutations are found in the four known MMR genes. There may be additional causative genes, mutations in known MMR genes that are not detected by current sequencing protocols (e.g., deep intronic mutations or promoter mutations) or epigenetic effects that remain to be discovered.^37^

## Availability of tumour testing and DNA testing for Lynch syndrome

The GeneTests Laboratory Directory (http://www.ncbi.nlm.nih.gov/sites/GeneTests/lab) lists 28 laboratories offering MSI analysis for Lynch syndrome. All of these laboratories test at least the 5 Bethesda guidelines markers; according to GeneTests, most use a panel of 10. Nine of the laboratories offer both tumour analysis by IHC and germline mutation testing. Approximately 50 labs are listed as offering DNA testing for *MLH1* and *MSH2* mutations (generally full sequence analysis plus testing for large deletions or duplications, but a few offering most restricted testing of specific exons). Smaller numbers of laboratories are listed for *MSH6* and *PMS2* mutation testing: approximately 40 and 25 respectively. 10 listed laboratories offer deletion testing of the *EPCAM* gene.

The GeneTests Laboratory Directory is not exhaustive and includes mostly US laboratories; Lynch syndrome diagnostic testing is also carried out by many additional commercial and hospital- or university-based diagnostic laboratories worldwide.

## Public health importance

If screening of all newly diagnosed endometrial cancer patients were to be undertaken, the incidence of endometrial cancer in the population will determine the number of women who will need to be offered tumour testing, and the expected proportion of these who have Lynch syndrome will determine the numbers who subsequently need surveillance for colorectal cancer (and possibly other Lynch syndrome-associated cancers). In the US, approximately 49,500 new cases of uterine cancer are expected in 2013, and 8,200 deaths from this cause.^38^ About 95% of uterine cancers are endometrial.^37^ Lynch syndrome accounts for about 2-4% of those diagnosed with endometrial cancer. Using the American Cancer Society figures for total diagnoses annually,^38^ the expected number of endometrial cancer diagnoses attributable to Lynch syndrome in 2013 is approximately 940-1880. Estimates of the prevalence of Lynch syndrome in the population range from approximately 1 in 3000^39^ to 1 in 370.^40^

Colorectal cancer (for which both the index case with Lynch syndrome and her relatives are at risk) is the fourth most common cancer in the US, with 142,820 new diagnoses and 50,830 deaths expected in 2013.^38^ In both men and women, it is the third-highest cause of cancer related deaths. Approximately 2-4% of colorectal cancer cases are attributable to Lynch syndrome, accounting for 2850-5700 of the expected incident cases in 2013.^41^

Estimated cancer risks for those with Lynch syndrome vary widely, even among studies that are either population-based or use statistical methods to correct for the ascertainment bias resulting from selection of patients from familial cancer clinics.^42^^,^ 43 ^,^ 44 ^,^ 45 ^,^ 46 ^,^ 47 Reported risks to age 70 for colorectal cancer range from 20-90% for men and 10-55% for women. Endometrial cancer risk estimates range from 15-55%, and risk of any Lynch syndrome cancer from 40-80% for men and 25-80% for women. Cumulative lifetime risks for other Lynch syndrome cancers are even more uncertain, but estimates generally do not exceed 15%.^46^^,^ 48 ^,^ 49 ^,^ 50 ^,^ 51 Some, but not all, of the variation is accounted for by different risk profiles for different MMR gene mutations: in general, risks for *MLH1 *or *MSH2 *carriers are higher, and age at onset of disease lower, than for those with mutations in *MSH6 *or *PMS2*.^52^^,^ 53

The cancer risks faced by those with Lynch syndrome may be compared with an average population risk to age 75 for colorectal cancer in the US of 3.0% in men and 2.3% in women, while women have a 2.0% risk of developing uterine cancer by age 75.^54^ Corresponding lifetime risks in the US are 5.3% and 4.9% for colorectal cancer in men and women respectively, and 2.6% for uterine cancer in women.^54^

## Published reviews, recommendations and guidelines

**Investigation of index endometrial cancer patients**

- The Society of Gynecologic Oncologists guidelines recommend that women at greater than 20-25% risk of Lynch syndrome should be offered genetic assessment.^55^ Risk is calculated on the basis of the revised Amsterdam criteria, or a diagnosis of synchronous or metachrononous endometrial or colorectal cancer before the age of 50, or a first or second degree relative with a known MMR mutation, or with evidence of a MMR defect by MSI or IHC analysis. The guidelines state that it is “reasonable” to offer genetic assessment to women whose risk is greater than 5-10%. The guidelines also acknowledge that a lower threshold for genetic referral may be reasonable in some circumstances; for example for women with very few female relatives or where there is adoption in their lineage. The strategy for “genetic risk assessment” is not specified.
- NCCN guidelines version 2.2012 recommend tumour testing when the patient meets Amsterdam or Revised Bethesda criteria, or has been diagnosed with endometrial cancer at age <50 years, or has known Lynch syndrome in the family.^56^ A testing strategy is specified based on initial tumour testing by IHC and/or MSI followed by germline DNA testing informed by the combination of tumour testing results.
- The National Society of Genetic Counselors and the Collaborative Group of the Americas on Inherited Colorectal Cancer joint practice guideline recommends that MSI and IHC analysis should be performed on endometrial tumours of patients meeting Amsterdam I or II or Bethesda criteria.^57^ Outline recommendations for optimum performance of MSI and IHC are included in the guideline.
- The Netherlands Association of Comprehensive Cancer Centres (ACCC) Guidelines on hereditary colorectal cancer include a recommendation that women with endometrial cancer diagnosed under the age of 50 should be referred to a clinical geneticist.^58^ For endometrial tumours, testing by both IHC and MSI is recommended, as well as methylation testing of the *MLH1 *promoter, to determine which women should be offered germline genetic testing.
- The “Mallorca group” of European experts recommends that all women diagnosed with endometrial cancer up to the age of 70 years should be offered tumour testing by MSI or IHC.^59^

**Genetic testing, surveillance and preventive options for people with Lynch syndrome**

There is general expert consensus that, when mutation testing has identified Lynch syndrome in an individual, germline genetic testing for the pathogenic mutation should be offered to his or her first-degree relatives (e.g. 23 ^,^ 56). Guidelines by the American Cancer Society, US Multi-Society Task Force and American College of Radiology state that genetic testing should be offered to first-degree relatives of those with a confirmed MMR mutation or, when the mutation is not known, if one of the first three of the modified Bethesda criteria is met.^60^

There is also broad consensus on surveillance and preventive options for colorectal cancer in people with Lynch syndrome. Guidelines published by professional associations and expert groups in the US and Europe (e.g. 59 ^,^ 60 ^,^ 61 ^,^ 62) and a systematic review by an independent expert group^63^ recommend colonoscopic surveillance every 1-2 years, beginning at age 20-25, or 10 years younger than the age of the youngest person diagnosed in the family (or 2-5 years before the earliest colon cancer if it is diagnosed at age <25 years).^56^ Lindor et al. suggest that screening may begin later (age 30) in those with *MSH6 *mutations.^63^

There is less consensus among professional guidelines on surveillance options for other Lynch syndrome-associated cancers. Some guidelines suggest screening for endometrial cancer and ovarian cancer by transvaginal ultrasound and endometrial biopsy every 1-2 years from age 30-35, while acknowledging a lack of convincing supportive evidence(e.g. 59 ^,^ 61 ^,^ 63 ^,^ 64). NCCN 2012 guidelines recommend that patients should be made aware that abnormal uterine bleeding warrants investigation, and state that surveillance options may be offered at the physician’s discretion.^56^ There is general consensus that prophylactic hysterectomy and bilateral salpingo-oophorectomy are of demonstrated benefit in reducing risk of endometrial and ovarian cancers and may be suggested as options for women aged 35 or over who have completed childbearing.^56^

For gastric cancer, small bowel cancer and urothelial cancer, some guidelines suggest that surveillance options should be considered, although clear supportive evidence is lacking.^56^^,^ 61 ^,^ 63

## Analytic validity of diagnostic tests

There is little direct evidence on the analytic validity of tests to diagnose Lynch syndrome in endometrial cancer patients. Tumour testing protocols for colorectal cancer specimens may not be optimal for endometrial tumours. Analytic validity may be affected by tumour sampling and preparation, by treatment the patient has undergone before the tumour is sampled (for example, there is some evidence from colorectal tumours that neoadjuvant therapy can affect MSI and IHC testing),^65^^,^ 66 and by variations in assay methods and competency among different laboratories.

** **MSI**

Current best practice, developed for testing colorectal tumours, is considered to include sample preparation by laser microdissection, use of a panel of at least 5 microsatellite markers, at least three of which should be mononucleotide markers, and ensuring a minimum proportion of 30% tumour cells in the sample analysed.^23^ Two highly polymorphic pentanucleotide markers can also be included to check for sample mix-up or contamination.^4^^,^ 67 Commercial test kits are available.

Many laboratories offering MSI testing participate in proficiency testing programmes (for example, the College of American Pathologists programme 68) and performance standards are assumed to be high if best practice is followed. In analyses of 646 tumour samples (of which 88% were colorectal tumours) Bartley et al. identified failure of DNA amplification in only 0.6% of the microsatellites analysed; in no case did the failure to amplify affect the designation of the tumour as MSS, MSI-H or MSI-L.^69^ A specific issue for MSI testing in endometrial tumours arises from the higher proportion of *MSH6 *mutations among endometrial cancer patients with Lynch syndrome (see below) and the fact that *MSH6*-deficient tumours frequently show an MSI-L and occasionally an MSS phenotype.^2^^,^ 70 Therefore an appropriate proficiency test should include performance in identifying the MSI-L phenotype in endometrial tumours.

**IHC**

Technical guidelines and proficiency testing for IHC are available in the US (for example, the College of American Pathologists programme)^68^ and in Europe (for example through the United Kingdom National External Quality Assessment Service).^71^ Most, if not all, proficiency testing programmes relate to the testing of colorectal tumours and specific schemes for endometrial tumours are not described. Clarke and Cooper mention an unpublished proficiency test for IHC involving 14 laboratories; they imply that the results showed high standards of performance but no details are given.^14^ However, some of the few publicly available results of IHC proficiency testing suggest some concerns. Nordic Immunohistochemistry Quality Control (NordiQC; www.nordiqc.org) publishes aggregated results from 80-90 participating laboratories for MMR protein IHC in colorectal tumour samples of known mutation status. For IHC of MLH1, MSH2 and MSH6, the percentage of submitted tests classed as optimal or good was 57%, 73% and 33% respectively; PMS2 is apparently not assessed by this programme. Substantial variation was found for antibody preparations from different commercial suppliers. Problems such as failure to recognise lack of an internal positive control, inter-observer variation (particularly among less-experienced pathologists) and difficulty in interpreting weak staining have been noted in several reports;^69^^,^ 72 ^,^ 73 these studies relate to colorectal tumours. There is little information about technical performance of IHC specifically in endometrial tumours, however Modica et al. claim that more apparent staining inadequacies are observed in endometrial than in colorectal tumours.^74^

**Methylation tests**

Insufficient information was found to enable evaluation of the analytical validity of methylation tests. There are several methylation sites in the *MLH1 *promoter but only those in the proximal C and D regions of the promoter correlate with silencing and loss of expression of MLH1,^67^ and some publications that report using methylation-specific PCR (MSP) of bisulphite-treated DNA to assay methylation do not specify which promoter region or regions were tested (e.g., 1). The MSP method is reportedly simple, sensitive, inexpensive and can be used on paraffin-embedded samples.^75^ Hampel et al. assayed the promoter H region by MSP and the D region by combined bisulphite restriction analysis.^2^ Testing of the D region was unsuccessful in 2 out of 118 tumours. Leenen et al. tested *MLH1 *promoter hypermethylation by MS-MLPA, a semi-quantitative method that analyses several promoter methylation sites simultaneously and is reportedly sensitive and reproducible.^67^ The proprietary kit they used, which contains probes specific for methylation sites in the MMR gene promoters, is available for research use only. Moline et al. report methylation testing by real-time PCR/fluorescence energy transfer at a commercial laboratory; no information on test performance is provided.^76^

**MMR gene sequencing**

The current clinical standard is direct gene sequencing combined with appropriate analysis (e.g., by MLPA) to detect large rearrangements in the four MMR genes and the *EPCAM *gene. It is unclear what the appropriate measures are for determining analytic validity. Analysis of the *PMS2 *gene is problematic because of the large number of pseudogenes. Improved methods of mutation detection in *PMS2 *have been described and reported to improve the sensitivity of mutation detection but the optimum method is currently unclear.^77^^,^ 78 ^,^ 79 ^,^ 80

Hampel et al. report failure of MLPA deletion analysis in approximately 15% of patients with MSI-positive tumours.^2^ Weissman et al. note that some MLPA kits for detection of *MSH2* deletions also encompass possible *EPCAM* deletions^57^ but data on the performance of this or other MLPA tests for *EPCAM* deletions (including the commercial MS-MLPA kit which can be used to detect methylation of the *MSH2* promoter)^67^ are not available.

New massively parallel “next-generation” sequencing technologies may eventually improve accuracy and throughput and decrease costs for mutation detection. For example, the Col Seq assay reportedly has 100% sensitivity for identifying pathogenic mutations in the 4 MMR genes and *EPCAM*, with 100% reproducibility between runs.^81^

## Clinical validity of diagnostic tests

Several studies were found that are relevant to the issue of tumour testing of unselected endometrial cancer patients. These studies (discussed below) were:

- A retrospective study by the OSU group on a large series of unselected endometrial cancer patients diagnosed between 1999 and 2003 (Hampel et al. with follow-up work reported by Mercado et al.);^2^^,^ 3 ^,^ 19
- A prospective study, reported by Backes et al., of 140 unselected endometrial cancer patients diagnosed at OSU in 2007 and 2008;^82^
- A retrospective study of 384 unselected endometrial cancer patients diagnosed at OSU from 2007-2009, reported by Backes et al.;^83^
- A prospective study by Leenen et al. of 179 consecutive endometrial cancer patients diagnosed up to the age of 70 in a multi-centre study in the Netherlands;^4^
- A prospective study by Lu et al. of 100 endometrial cancer patients diagnosed under the age of 50;^1^
- A retrospective study by Berends et al. of 58 endometrial cancer patients diagnosed under the age of 50;^84^
- A prospective study by Goodfellow et al. of 441 unselected endometrial cancer patients; molecular analysis, and mutation testing for *MSH6 *mutations, was carried out on a subset of 100 of these patients.^20^

Not all of these studies contained sufficient information to contribute to quantitative estimation of the clinical validity of tumour testing, defined as the performance of the test(s) in identifying patients with a pathogenic germline mutation in an MMR gene or the *EPCAM* gene: in three of the studies, germline mutation testing was carried out only on some or all of those patients with tumour test results suggestive of Lynch syndrome, and not on patients with negative tumour test results.^4^^,^ 82 ^,^ 83 Moline et al. have recently reported the results of implementing a tumour testing programme for 245 endometrial cancer patients at Cleveland Clinic. However, both the inclusion criteria for tumour testing and the tumour testing protocol changed over the period reported, making it difficult to draw quantitative conclusions from the pooled results.^76^

**MSI**

The ideal study would comprise both MSI tumour testing, and germline DNA testing for mutations in the *MLH1*, *MSH2*, *MSH6*, *PMS2 *and *EPCAM *genes, in a large series of consecutive, unselected endometrial cancer patients. No such studies were found. However, the study by the OSU group used broad inclusion criteria for germline testing (see Table 1 footnotes), with test sensitivity and specificity apparently calculated on the assumption that the overall testing strategy identified all mutation carriers.^2^^,^ 3 ^,^ 19 This study also included analysis of a sample of clinic-based cases and their relatives.

Some studies were also identified in which MSI was analysed only in tumours from patients with known MMR gene mutations.^11^^,^ 70 ^,^ 75 Although not ideal, as no mutation-negative individuals were tested, investigators were not blind to mutation status, and the populations are not representative of an unselected population of endometrial cancer patients, these studies were included to contribute to estimates of test sensitivity.

As shown in Table 1, estimates of the sensitivity of MSI analysis range from 77-100%, with specificity 38-81% overall, and 69-81% in population-based studies. The positive predictive value in the population-based OSU study was 9%; in the two studies restricted to patients under the age of 50, it was higher (20% and 32%). The negative predictive value was 97-100%. However, comparisons are problematic, and conclusions difficult to draw, due to heterogeneity among studies (e.g. different populations; different markers and criteria for MSI analysis; different genes included in germline DNA testing; different mutation detection methods). The EGAPP working group noted similar difficulties in evaluating studies on colorectal tumours.^21^^,^ 23

**Table 1. Clinical validity of MSI testing:** 

Study	Mutation carriers (total MSI tested)	MMR genes tested	MSI panel (no. mononuc. markers)	MSI criterion	Sens.	Spec.	PPV	NPV
Mercado^a^ 19	13 (560)	*MLH1*, *MSH2*, *MSH6*, *PMS2*	5-6 (2)	MSI-H + L	0.92	0.78	0.09	1.00
Mercado^b ^ 19	16 (24)	*MLH1*, *MSH2*, *MSH6*, *PMS2*	5-6 (2)	MSI-H + L	1.0	0.38	0.76	1.00
Lu^c ^ 1	18 (95)	*MLH1*, *MSH2*, *MSH6*	6 (3)	MSI-H	1.0	0.81	0.32	1.0
Berends^d ^ 84	5 (57)	*MLH1*, *MSH2*, *MSH6*	5 (2)	MSI-H	0.80	0.69	0.20	0.97
Goodfellow^e ^ 20	7 (100)	*MSH6*	5 (2)	MSI-H	1.0	-	-	-
Ryan^f ^ 11	20 (20)	*MLH1*, *MSH2*, *MSH6*	5-10 (2-3)	Unclear	0.90	-	-	-
de Leeuw^g ^ 70	31 (31)	*MLH1*, *MSH2*, *MSH6*	5 (2) 24-40 (8)	MSI-H + L MSI-H + L	0.77 0.97	-	-	-
Kuismanen^h^ 85	57 (57)	*MLH1*, *MSH2*	12 (10)	MSI-H + L	0.77	-	-	-

Sens., sensitivity; Spec., specificity; PPV, positive predictive value; NPV, negative predictive value

^a ^MSI analysis on tumour samples for 560 of 563 unselected patients. IHC for all MSI tumours, or for women diagnosed at <50 years, or for women with synchronous or metachronous colorectal cancer and endometrial primaries, or with a first degree relative diagnosed with endometrial or colorectal cancer at any age. 223 MSS tumours also evaluated by IHC. Subjects with MSI-H or MSI-L and/or abnormal IHC underwent germline DNA testing. Germline testing by direct sequencing (*MLH1*, *MSH2*, *MSH6*), and MLPA for all 4 genes.

^b ^Patients from family cancer clinics and their affected and unaffected relatives.

^c ^Consecutive endometrial cancer patients diagnosed <50y. All tested by MSI and germline mutation analysis by full sequencing and large deletion analysis.

^d ^Endometrial cancer patients diagnosed <50y. All tested by MSI and germline mutation analysis (DGGE with sequence variants verified by direct sequencing; MLPA for large deletions). Only those with ≥2 unstable markers were classified as MSI-H.

^e ^Those tested for germline mutations were a subset of 441 unselected patients: all those with MSI-H and unmethylated *MLH1* promoter (30), all those with MSI-L (10), 30/92 with MSI-H and methylated *MLH1* promoter, and 30/304 MSS. Mutation analysis by SSCV to detect variants which were then confirmed by direct sequencing.

^f ^Patients from family cancer clinics. Initial MSI with 5 markers (Bethesda panel) plus additional analysis with 10-marker panel (3 mononucleotide markers) for MSI-L tumours. Germline testing by direct sequencing and MLPA; no details given.

^g ^Retrospective study on known mutation carriers with endometrial tumours (23 carcinomas, 8 hyperplasias), from families fulfilling Amsterdam I criteria

^h ^Retrospective study on 57 tumours from known mutation carriers from Lynch syndrome families with 8 different *MLH1* mutations and one *MSH2* mutation.

A common finding in several studies of MSI in Lynch syndrome endometrial tumours has been a lower frequency of MSI in tumours from *MSH6 *mutation carriers. For example, 3 of the mutation carriers identified in the retrospective OSU studies had tumours that were MSI-L or MSS: all 3 of these patients had *MSH6 *mutations.^2^^,^ 3 De Leeuw et al. found that tumours from 12 *MSH6 *mutation carriers showed a low level of MSI across a panel of 30-40 markers. Only 4 were classified as MSI-H by Bethesda guidelines panel (5 marker) criteria, and tumours showed instability only with mononucleotide markers.^70^ Berends et al. found that the one mutation carrier whose tumour was not identified as MSI-H with the Bethesda guidelines panel was an *MSH6 *carrier.^84^

**IHC**

Table 2 shows results for clinical validity of IHC analysis in 3 population-based and 4 clinic-based studies, 3 of which were case-only studies.^1^^,^ 11 ^,^ 19 ^,^ 70 ^,^ 84 ^,^ 85 Overall sensitivity for 3 or 4 MMR proteins ranged from 86-100%, and specificity from 48-81% (though the range for population-based studies was narrower: 59-81%). As for MSI analysis, the positive predictive value of IHC in the OSU study was lower (10%) than in the studies restricted to patients under 50 years (20% and 38%). The negative predictive value in all three studies was 100%. As with MSI analysis, it is difficult to draw overall conclusions because of the heterogeneity among studies. Clarke and Cooper state an overall sensitivity of 96-100% for the four MMR protein markers in an MMR-IHC proficiency test “using tissue microarrays of carcinomas of known germline MMR mutation status”.^14^ The rest of their paper relates to endometrial cancer, but the authors do not specify whether the proficiency testing results (which are described as unpublished) relate to endometrial tumours, or colorectal tumours, or both, or whether the tumours were from unselected cases or cases ascertained through family cancer clinics. Some pathogenic missense variants of MMR proteins escape detection by IHC, lowering the sensitivity of the test;^29^^,^ 84 ^,^ 85 ^,^ 86 this was the case for two pathogenic mutations in the OSU study.^2^^,^ 84 It has been reported that IHC testing of MSH6 and PMS2 alone in Lynch syndrome tumours is as effective as a four-antibody test,^87^ and this two-antibody test is in routine use in the endometrial tumour screening programme at the Cleveland Clinic;^76^ however the clinical validity of the test does not appear to have been formally evaluated.

**Table 2 Clinical validity of IHC testing:** 

Study	Mutation carriers (total IHC tested)	MMR genes/proteins tested	Sens.	Spec.	PPV	NPV
Mercado^a^	13 (352)	MLH1, MSH2, MSH6, PMS2	0.86	0.67	0.10	1.00
Mercado^b^	51 (80)	MLH1, MSH2, MSH6, PMS2	0.94	0.48	0.80	0.79
Lu	9 (99)	MLH1, MSH2, MSH6	1.00	0.81	0.38	1.00
Berends^c^	5 (51/36)	MLH1, MSH2, MSH6	1.0	0.59	0.21	1.00
Ryan^d^	23 (23)	MLH1, MSH2, MSH6	0.91-1.0	-	-	-
de Leeuw^e^	31 (31)	MLH1, MSH2, MSH6	0.97	-	-	-
Kuismanen	18 (18)	MLH1, MSH2	1.0			

For references for studies, see legend to Table 1.

The senstivity (Sens.), specificity (Spec.), positive predictive value (PPV) and negative predictive value (NPV) relate to the ability of the test to give a result consistent with the germline mutation.

^a^ Population-based study

^b ^Clinic-based cohort

^c ^51 tumours were tested for MLH1 and MSH2 and 36 were tested for MSH6.

^d ^The sensitivity of 1.0 includes all tumours with results classed as “definite” or “equivocal”. For those classed as “definite”, sensitivity was 0.96. Both “equivocal” tumours were from *MSH2* mutation carriers.

^e ^Note that staining for 6/8 MLH1-deficient tumours was described as “weak” but classed as negative in this study.

**Selection of patients with abnormal tumour testing results for referral for germline DNA testing**

Two of the population-based studies of unselected endometrial cancer patients contained information relevant to the clinical validity of methylation testing for detecting sporadic tumours, but the data were incomplete.^1^^,^ 2 ^,^ 3 Lu et al. tested for *MLH1 *promoter hypermethylation by the MSP method in 13 patients (from their total series of 99) whose tumours were MSI-H and showed loss of MLH1 by IHC, or had uncertain IHC results. However, it is not clear which *MLH1 *promoter region was assayed, and methylation analysis was apparently not performed in the one patient with a germline *MLH1 *mutation.^1^ Twelve of the 13 tumours were methylation-positive; the one negative result was for a MSI-H tumour for which IHC testing did not work. No known pathogenic mutations were found in any of these tumours, though variants of uncertain significance were found in two patients (one in *MLH1 *and one in *MSH2*). If these variants are assumed to be non-pathogenic, the sensitivity of methylation testing for detecting sporadic tumours after IHC and MSI testing in this study was 92%.

The OSU study reported by Hampel et al. is complicated by the fact that they assayed two regions of the *MLH1 *promoter, only one of which (the D region) is reported in other publications to be associated with *MLH1 *silencing. They also used a different method for each region. In addition, one *MLH1 *mutation carrier reported in the later publication by Mercado et al.^19^ is not included in the earlier publications on the OSU cohort;^2^^,^ 3 the MSI, IHC and *MLH1 *promoter methylation status for this patient’s tumour are unclear. Methylation at the D region detected 85% of the sporadic tumours that were both MSI-H and lacked MLH1 protein.^2^

Leenen et al. found *MLH1* promoter methylation in 31 of 32 tumours that were MSI-H and lacked MLH1 expression (18% of the total tumours tested), and considered the test to be robust enough to exclude these patients from germline DNA testing.^4^ The clinical validity of the methylation test cannot be confirmed from this study. Zauber et al. tested MSI and methylation status by MS-MLPA in 101 unselected endometrial cancer patients under 50 years and 112 older than 50 years.^88^ The combination of MSI-H and unmethylated *MLH1 *promoter indicated presumptive Lynch syndrome in 13% of the younger women and 5% of those over 50 years, but mutation testing was not carried out in this study.

The OSU group suggest alternative criteria, instead of methylation analysis, to prompt referral for germline DNA testing after initial tumour testing by IHC: all those with loss of MSH2/MSH6, and those with loss of MLH1/PMS2 who are under 60 or have a concerning family history. (In those over 60, loss of MLH1/PMS2 and no concerning family history is considered to indicate likely *MLH1 *promoter methylation and therefore a sporadic cancer).^13^^,^ 82 ^,^ 83 All 13 mutation carriers identified in the first two publications on the OSU cohort met these criteria; no published information was found on the subsequently identified *MLH1 *mutation carrier.^2^^,^ 3 11% of the OSU prospective series of 140 patients met the referral criteria (about half of the number who showed loss of at least one MMR protein by IHC); however, no mutations were found in the two patients who accepted germline genetic testing.^82^ In the subsequent retrospective study reported by Backes et al., the referral criteria were found to predict an MMR gene mutation in 3 out of 8 patients who underwent genetic testing (out of a total of 27 referrals).^83^ If the same referral criteria had been applied in the study by Leenen et al., one of 11 patients with likely Lynch syndrome on the basis of IHC and MSI testing would not have been referred for genetic counselling; as this patient (whose tumour was negative for *MLH1 *promoter hypermethylation) declined germline genetic testing, their mutation status is not known.^4^

For centres where tumour testing for all endometrial cancer patients is not considered feasible, the OSU group suggest restricting tumour testing to patients under the age of 60.^13^ However, Leenen et al. note that 5 of the 11 patients referred for genetic consultation in their study were over the age of 60; of these, 3 were found to be mutation carriers and one declined testing. None fulfilled Amsterdam or Bethesda criteria.^4^

Kwon et al. suggest referral of patients with abnormal IHC results and who have one affected first-degree relative.^89^ However, the clinical sensitivity of this criterion in unselected patients is likely to be low (and see further discussion below).^1^^,^ 4 ^,^ 19

**Age at diagnosis and MMR gene mutations in endometrial cancer patients with Lynch syndrome**

Table 3 shows the mutation spectrum, and mean or median age at diagnosis, of Lynch syndrome endometrial cancer in 4 population-based studies and a sample of 3 clinic-based studies. In the two population-based studies not restricted to patients under the age of 50, pathogenic mutations were identified in 2.5% and 3.9% of patients respectively.^4^^,^ 19 The most striking difference between these two studies and those either in clinic-based cohorts^11^^,^ 19 ^,^ 46 or young patients^1^^,^ 84 is in the proportion of *MSH6 *mutation carriers: 64% in the OSU study^19^ and 87% in the Netherlands study.^4^ The average age at diagnosis in the OSU and Netherlands studies is similar to that for *MSH6 *carriers in clinic-based studies and is older than that for *MLH1 *or *MSH2 *mutation carriers. The relatively high frequency of *MSH6 *mutations and older age at onset appears to be characteristic of mutation carriers from unselected endometrial cancer populations not restricted by age at diagnosis (or with cut-off at a late age). For example, Goodfellow et al. found a minimum frequency of 1.6% *MSH6* mutations in 441 unselected endometrial cancer patients; 71% of those with *MSH6 *mutations were over 50.^20^ Devlin et al. found 6 *MSH6 *mutations (4 truncating mutations and 2 possibly pathogenic missense mutations) in 105 unselected patients.^90^ (No analysis was carried out for the other three MMR genes in either study.) In a large study of *MSH6 *mutation carriers, Baglietto et al. found a mean age of onset for endometrial cancer of 51.^53^

In the Lynch syndrome tumour screening programme reported by Moline et al., Lynch syndrome was confirmed by mutation testing in 8 patients, of whom 2 had mutations in *MLH1*, 2 in *MSH2*, 2 in *MSH6 *and 2 in *PMS2*.^76^ Three additional patients had tumour testing IHC results strongly suggestive of Lynch syndrome (lack of MSH2/MSH6 by IHC) but no mutations could be found. These patients were considered likely to have Lynch syndrome, giving a total of 11 definite/likely cases out of 245 patients tested (4.5%). As mentioned previously, comparisons with reported research studies are difficult due to the pooling of patients selected by different criteria.

In the Netherlands studies, no pathogenic mutation could be found in 30% (3/10) of individuals whose tumours were MSI-H, methylation-negative for the *MLH1 *promoter, and had IHC results indicating the loss of a MMR protein.

**Table 3. Age at diagnosis and MMR gene mutation spectrum in endometrial cancer patients with Lynch syndrome:** 

Study	Mutation carriers	Age at diagnosis (mean or median)	*MLH1* No. (%)	*MSH2* No. (%)	*MSH6* No. (%)	*PMS2* No. (%)
Mercado^a^ 19	14	53	2 (14%)	3 (21%)	9 (64%)	0 (0%)
Leenen (patients <70y)^b^ 4	7	59	0 (0%)	0 (0%)	6 (87%)	1 (13%)
Lu (patients <50y)^c^ 1	9	42	1 (11%)	7 (78%)	1 (11%)	n.d.
Berends (patients <50y)^84^	5	45	1 (20%)	3 (60%)	1 (20%)	n.d.
Ryan^11^	76	47 (overall) 49 (*MLH1*) 46 (*MSH2*) 51 (*MSH6*)	18 (24%)	50 (66%)	8 (10%)	n.d.
Mercado^19^	80	48	31 (39%)	40 (50%)	9 (11%)	0 (0%)
Bonadona^46^	182	49 (*MLH1*) 48 (*MSH2*) 55 (*MSH6*)	72 (39%)	87 (48%)	23 (13%)	n.d.

^a ^12 mutations of unknown significance (mostly point mutations or small insertions/deletions) reported: 2 in *MLH1*, 3 in *MSH2* and 7 in *MSH6*.

^b ^No mutation found in 3 patients with IHC results suggesting loss of expression, and 1 patient declined germline testing

***EPCAM* gene analysis**

*EPCAM *gene analysis may be considered in cases where a tumour shows absent *MSH2 *expression but no germline *MSH2 *mutation is found.^91^^,^ 92 Neither the OSU group nor the Netherlands group tested for *EPCAM *mutations. Further research is needed to clarify the contribution of *EPCAM *mutations in endometrial cancer. Kempers et al. found that the endometrial cancer risk for *EPCAM *mutation carriers depends strongly on the location of the deletion in the *EPCAM *gene: an average cumulative risk of 12% to age 70 falls to almost zero for deletions located far upstream of the *MSH2 *promoter, but rises to 30% for deletions extending closer to the promoter (estimates not corrected for ascertainment bias).^92^

**Interpretation of DNA test results**

Based on experience with colorectal cancer, it has been estimated that in approximately 7% of at-risk individuals who have genetic testing for MMR gene mutations, a variant of uncertain significance is found.^40^ The interpretation of DNA test results may be aided by databases of Lynch syndrome mutations such as the InSiGHT (International Society of Gastrointestinal Hereditary Tumours) database (www.insight-group.org, which merged several previous databases and includes information on variants of unknown significance (however, this database relates primarily to Lynch syndrome colorectal cancer).^93^ A multivariate model has been developed to aid classification of missense variants in *MLH1 *and *MSH2*.^94^ Functional assays may also aid in determining pathogenicity.^95^

## Clinical utility

The clinical utility of screening all incident endometrial cancer cases for Lynch syndrome relates to the likelihood that screening will lead to improved health either in the index case or in her relatives.

**Index case**

In order to demonstrate clinical utility for the index case, there needs to be adequate evidence that tumour testing and (if indicated) germline DNA analysis are acceptable to women and do not themselves cause significant harms, and that a diagnosis of Lynch syndrome (a) indicates specific treatment for Lynch syndrome endometrial cancer that decreases mortality or morbidity from the condition and/or (b) indicates surveillance and/or preventive options that are acceptable and decrease mortality and/or morbidity from other Lynch-syndrome associated cancers.

**Acceptability of tumour testing and germline DNA analysis**

Limited published information is available on the acceptability of tumour testing, genetic counselling and germline DNA analysis among unselected women diagnosed with endometrial cancer. Arguments for and against reflex tumour testing without explicit consent have been discussed for colorectal cancer patients and similar considerations apply in the case of endometrial cancer patients.^21^

Backes et al. found a low level of acceptance of germline DNA testing among endometrial cancer patients referred for genetic counselling (13% of those offered referral in a prospective study and 30% in a retrospective study).^82^^,^ 83 Kuppermann et al. also suggest that recognition of the value of genetic testing may not be high among unselected patients.^96^ In the Cleveland Clinic programme, 32 out of 42 patients with abnormal tumour test results (76%) accepted referral for genetic counselling and 28 of those accepted an offer of genetic testing.^76^ In the Netherlands study by Leenen et al., 10 0f 11 patients with IHC, MSI and *MLH1 *promoter methylation results suggestive of Lynch syndrome consented to germline DNA testing.^4^ Cultural differences between European and US patients, and differences in health insurance arrangements, may affect the acceptability of genetic testing. Backes et al. found that concerns about insurance coverage, and patients’ underestimation of their cancer risk, were important factors in limiting interest in genetic investigation.^83^ An emphasis on information and explanation, for physicians as well as patients, is important.^4^^,^ 83

**Treatment of Lynch syndrome endometrial cancer**

Lynch syndrome endometrial cancers display some morphological and histological differences from sporadic cancers (summarised by Clarke and Cooper).^14^ However, no information was found to suggest that treatment of endometrial cancer for women with Lynch syndrome differs from treatment for sporadic endometrial cancer.

**Cancer risks and surveillance**

Endometrial cancer is the first malignancy in approximately 50% of women with Lynch syndrome.^7^^,^ 8 Women remain at risk of subsequent colorectal cancer and, to a lesser extent, of other Lynch-associated cancers. Recently, Win et al. have estimated cancer risks after endometrial cancer for women with Lynch syndrome, using data for 127 women from the international Colon Cancer Family Registry.^97^ The median age of endometrial cancer diagnosis in this population was 46. The major risk was found to be for colorectal cancer, with 10- and 20-year cumulative risks of 20% (95% CI 13%-28%) and 48% (95% CI 35%-62%) respectively, and a standardised incidence ratio (SIR), compared to the general population, of 39.9 (95% CI 27.2-58.3). The median time from endometrial cancer diagnosis to colorectal cancer diagnosis was 11 years. The study had insufficient power to fully stratify cancer risks by MMR gene mutation; however, a significant difference was nevertheless found between the SIR for colorectal cancer in *MSH6 *mutation carriers (SIR=4.46, 95% CI 0.00-24.2) and either *MLH1 *(SIR=38.7, 95% CI 19.5-70.2) or *MSH2 *(SIR 58.5, 95% CI 36.0-98.4) mutation carriers.

The difference in colorectal cancer risk between *MSH6 *and *MLH1*/*MSH2 *mutation carriers is consistent with data from studies on the cumulative risk of colorectal cancer in people with Lynch syndrome.^42^^,^ 43 ^,^ 44 ^,^ 45 ^,^ 46 ^,^ 47 As shown in Figures 1 and 2, in female *MLH1 *and *MSH2 *mutation carriers cumulative risk to age 70 ranges from 20-50%, with risk increasingly more sharply between ages 30-60 than at later ages. In female *MSH6 *and *PMS2 *mutation carriers, cumulative risk of colorectal cancer rises from 2-3% at age 50 to 20% at age 80. (Note that, for clarity, confidence intervals have not been shown for the studies illustrated in Figures 1-5. In all studies they are broad, particularly at older ages where numbers of cases are small.)

**Cumulative risk of colorectal cancer in women carrying MLH1 or MSH2 mutations:**
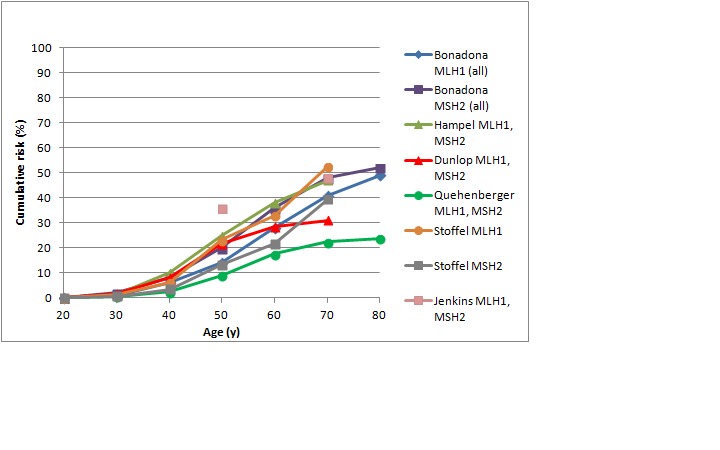
Studies shown: Bonadona,^46^ Hampel,^42^ Dunlop,^43^ Quehenberger,^44^ Stoffel,^47^ Jenkins^45^

**Cumulative risk of colorectal cancer in women and men with MSH6 or PMS2 mutations:**
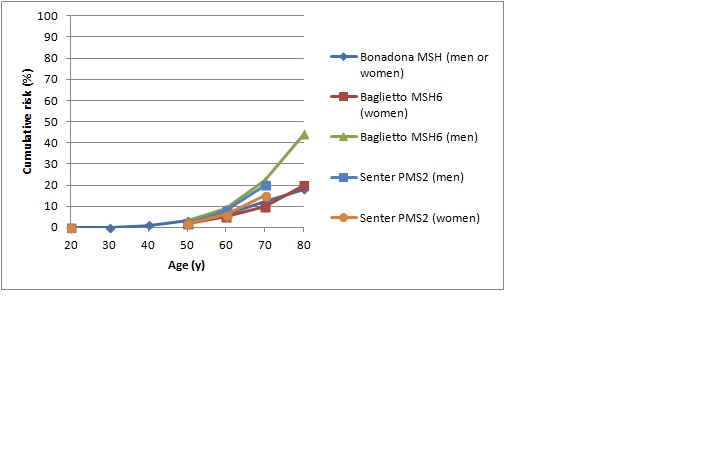
Studies shown: Bonadona,^46^ Baglietto,^53^ Senter^52^

As mentioned previously, colonoscopy every 1-2 years has been shown to reduce morbidity and mortality from colorectal cancer in people with Lynch syndrome. Studies in the United States and Germany report approximately 80% compliance with these recommendations in mutation carriers or those fulfilling Amsterdam or Bethesda criteria for risk of Lynch syndrome;^98^^,^ 99 however compliance was only about 60% in two studies in Italy and Spain.^100^ Psychological evaluations of the effects of risk reducing interventions are few but those that have been carried out suggest that appropriate advice and information help to improve knowledge and reduce anxiety (reviewed in 100)

The risks of non-colorectal cancers in women with Lynch syndrome who have had endometrial cancer are difficult to assess. Win et al. found increased 10- and 20-year cumulative risks for cancers of the urinary tract (kidney, renal pelvis or ureter) [2% (95% CI 0-5%) and 11% (95% CI 3-20%)], urinary bladder [1% (95% CI 0-4%) and 9% (95% CI 2-17%)] and breast [5% (95% CI 1-10%) and 11% (95% CI 4-19%)].^97^ Increased risks were also observed for small intestine and pancreatic cancer, though numbers of cases were small and confidence intervals for SIRs very large. In studies on cumulative lifetime risks for Lynch syndrome-associated ovarian, stomach, small intestine and biliary tract cancers, estimates vary so widely that few conclusions can be drawn.^44^^,^ 46 ^,^ 48 ^,^ 49 ^,^ 50 ^,^ 51 ^,^ 52 ^,^ 53 For women, the most significant risks from these studies appear to be for ovarian cancer and, in some recent studies, breast cancer. (see Table 3 in the paper by Win et al. at http://jco.ascopubs.org/content/30/9/958/T3.expansion.html);^101^ however, a recent systematic review from the same research group found that current evidence at the population level for an increased risk of breast cancer in Lynch syndrome was inconclusive.^102^

Although there are no surveillance interventions of proven benefit for non-colorectal Lynch syndrome cancers, urinalysis with cytology every 1-2 years beginning age 25-35 and upper gastrointestinal endoscopy every 1-2 years beginning age 30-35 are often recommended by physicians as surveillance regimes for upper urinary tract and stomach cancer respectively (reviewed in 6). In view of the recent evidence that breast cancer risk may be elevated in women with Lynch syndrome,^97^^,^ 101 enhanced surveillance for breast cancer may also be warranted, though Win et al. comment that risk may not reach the threshold lifetime risk of 20% recommended by the American Cancer Society for breast screening by MRI.^97^

**Preventive options**

Partial or subtotal colectomy is recommended only for individuals with Lynch syndrome who have been diagnosed with colorectal cancer, to reduce the risk of metachronous cancer.^59^ It is therefore not an appropriate option for women with Lynch syndrome ascertained through diagnosis of endometrial cancer, unless colorectal cancer is also present.

There is some recent evidence that chemoprophylaxis with aspirin can reduce the risk of colorectal cancer in individuals with Lynch syndrome.^103^^,^ 104 Benefit was not observed after 29 months of treatment but was found on longer-term follow-up (56 months). Further research is needed to confirm these findings and determine optimum aspirin dosage, as well as to investigate any adverse effects.^105^ No information is available with regard to whether the protective effect of aspirin would also apply to women who have had endometrial cancer.

For other Lynch-associated cancers, there are no preventive interventions of proven benefit except for ovarian cancer, where bilateral salpingo-oophorectomy has been shown to reduce risk and to be cost-effective.^106^^,^ 107 ^,^ 108 This may be an appropriate option for some women with Lynch syndrome identified through screening endometrial cancer patients, if they have not already undergone oophorectomy in conjunction with hysterectomy to treat their cancer. A preliminary report suggests that oral progestins may reduce the risk of endometrial cancer in women with Lynch syndrome.^109^

Overall, the clinical utility of Lynch syndrome testing for the index case depends on her age and the MMR gene mutated: the net benefit is lower for those diagnosed at older ages and with less-penetrant *MSH6 *mutations. To date, women with these features are the majority of those diagnosed through screening unselected endometrial cancer patients though the number of studies is small. Taken together, these findings suggest that careful age- and mutation-specific genetic counselling is likely to be essential for women with endometrial cancer who are found to have Lynch syndrome, to assist them in understanding their future risk of cancer and in weighing the risks and benefits of surveillance.

**Cost-effectiveness (index case)**

Resnick et al. carried out a cost-effectiveness analysis comparing triage strategies for identifying endometrial cancer patients with Lynch syndrome.^30^ They found that, relative to strategies of MMR gene sequence analysis for all women with endometrial cancer, for all diagnosed under age 60, or for all with endometrial cancer who meet Amsterdam criteria, the OSU strategy of IHC tumour analysis for all, followed by genetics referral and single-gene analysis except for those aged over 60 with loss of MLH1/PMS2 and no concerning family history, had an incremental cost-effectiveness ratio (ICER) of $13,812 per additional case detected, and would detect 858 of the estimated 920 incident Lynch syndrome endometrial cancer patients annually. The costs of subsequent surveillance and preventive interventions were not analysed.

Kwon et al. used a Monte Carlo simulation analysis to compare the costs and benefits of six different strategies for testing women with endometrial cancer for Lynch syndrome, assuming that all those identified would undertake risk-reducing colonoscopy every 1-2 years, and that this surveillance would reduce the risk of colorectal cancer from 40% to 15%.^89^ They concluded that the ICER for testing all women with endometrial cancer by initial IHC triage followed by germline DNA testing in those testing positive by IHC was unfavourable, at approximately $650,000 per life year gained, but that testing all those with at least one first-degree relative with a Lynch-syndrome associated cancer diagnosed at any age was cost-effective, with an ICER of approximately $9,000 per life year gained relative to the most inexpensive strategy (testing all women with endometrial cancer under the age of 50 who have at least one affected first-degree relative). The input data used by Kwon et al. come from a variety of sources, most of which relate to patients ascertained through family cancer clinics. Their assumptions on the extent of risk reduction, and the sensitivity of restricting germline testing to women with at least one affected first-degree relative, may not be valid for women identified by screening all incident endometrial cancer patients. For example, the family history restriction would exclude 9/11 of those referred for germline genetic testing in the study by Leenen et al., and 5/7 mutation carriers.^4^

**Relatives of the index case**

In order to demonstrate clinical utility in relatives of the index case, there needs to be sufficient evidence that targeted germline DNA testing is acceptable to these individuals, and that effective and acceptable interventions are available to enable them to reduce their risk of Lynch syndrome-associated cancers.

Risks of colorectal cancer and other non-uterine Lynch syndrome cancers in women have already been discussed (risks are not significantly affected by whether a woman has previously had endometrial cancer).^97^ For female relatives of the index case, there is the additional consideration that the diagnosis of Lynch syndrome may be made at a younger age; for young women, risks of colorectal cancer may be substantial for *MLH1 *or *MSH2 *mutation carriers (Fig 1).

Figures 3 and 4 show cumulative risks of endometrial cancer for *MLH1 *and *MSH2 *carriers,^42^^,^ 43 ^,^ 44 ^,^ 46 ^,^ 47 and *MSH6 *and *PMS2 *carriers,^46^^,^ 52 ^,^ 53 respectively. For *MLH1 *and *MSH2 *carriers, the risk profile is similar to that for colorectal cancer, with a risk to age 70 of 20-50%. For *MSH6 *or *PMS2 *carriers, risk is low to about age 50, rising to 15-25% by age 70 and possibly higher by age 80. Win et al. found overall 5- and 10-year cumulative risks for endometrial cancer of 2.84% and 9.84% respectively (see Table 3 at http://jco.ascopubs.org/content/30/9/958/T3.expansion.html).^101^

**Cumulative risk of endometrial cancer in MLH1 and MSH2 mutation carriers:**
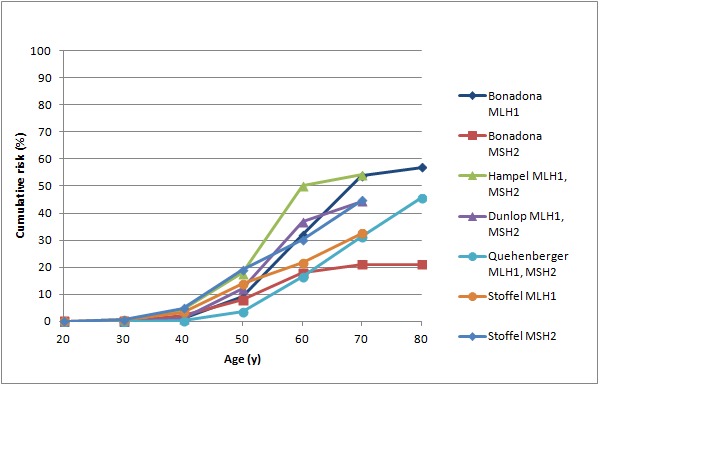
Studies shown: as for Figure 1

**Cumulative risk of endometrial cancer in MSH6 and PMS2 mutation carriers:**
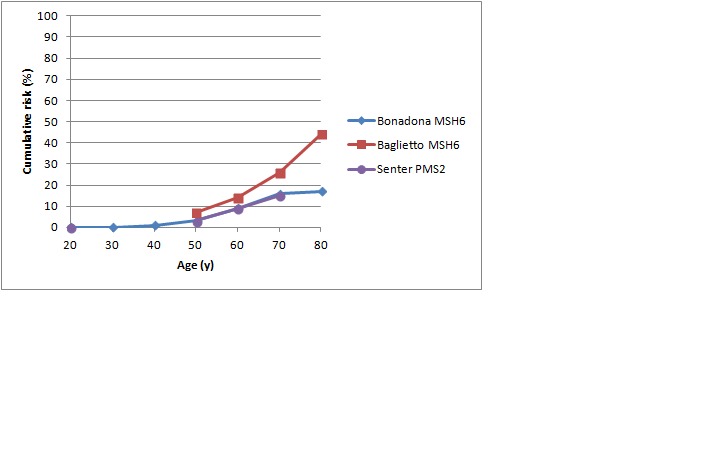
Studies shown: as for Figure 2

Figure 5 shows cumulative risks of colorectal cancer in men for *MLH1 *and *MSH2 *carriers.^42^^,^ 43 ^,^ 44 ^,^ 45 ^,^ 46 ^,^ 47 The range of estimates to age 70 is very high: from less than 30% to over 90% in different studies. Risks for colorectal cancer in male *MSH6 *or *PMS2 *carriers are shown in Figure 2.^46^^,^ 52 ^,^ 53 The risk for *PMS2 *mutation carriers is about 15%; for *MSH6 *mutation carriers, the two available studies found similar risks to age 70 (approximately 15-20%) but the large study by Baglietto et al. found that risk increased substantially between age 70 and 80.^53^

**Cumulative risk of colorectal cancer in men with MLH1 or MSH2 mutations:**
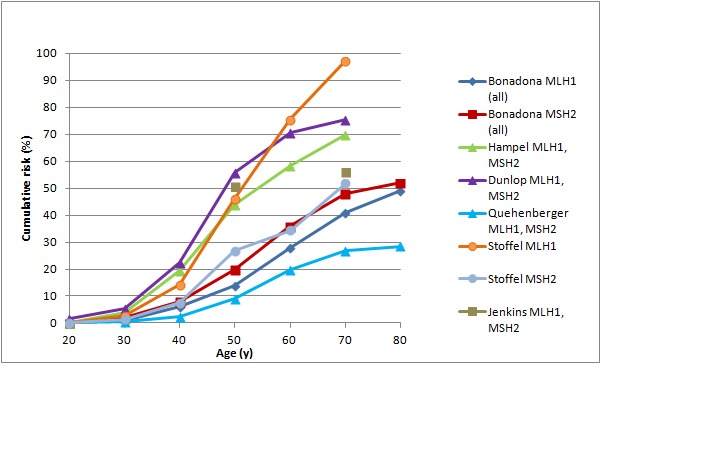
Studies shown: as for Figure 1

As discussed above, there is adequate evidence that surveillance by regular colonoscopy is effective in reducing risk of colorectal cancer in individuals with Lynch syndrome. The current recommendation is that screening should begin at age 20-25, or 10 years younger than the age at diagnosis of the youngest person diagnosed in the family. For families ascertained by diagnosis of an endometrial cancer patient with an *MSH6 *mutation, this might suggest delaying the start of screening to age 30-35 unless colorectal cancer cases in the family suggest otherwise.^63^

For endometrial cancer, there is some evidence that endometrial biopsy every 1-2 years, either alone or in combination with transvaginal ultrasound, is effective in lowering risk by early detection of cancer (reviewed in 6 ^,^ 110), but a recent systematic review concluded that current evidence is insufficient to enable evidence-based decisions, and that surveillance does not appear to lower risk of ovarian cancer.^64^ Given the very low risks in *MSH6 *or *PMS2 *carriers before middle age, if screening is undertaken it may be reasonable to start at the upper end of the suggested age band in these women.^63^ As a preventive intervention for endometrial and ovarian cancer, total hysterectomy and bilateral salpingo-oophorectomy has been shown to reduce risk,^106^ and to be acceptable to women diagnosed with Lynch syndrome mutations who have completed child-bearing.^111^

Jarvinen et al. found that, over an 11-year follow-up period, there was no difference in cancer mortality between individuals with Lynch syndrome undergoing risk-reducing surveillance and preventive interventions, and mutation-negative members of the same families, suggesting that current surveillance strategies are effective.^112^ In a large prospective study, Win et al. have found that mutation-negative members of Lynch syndrome families have cancer risks similar to those of the overall population and therefore do not need enhanced surveillance.^101^

Overall, clinical utility to relatives of an index Lynch syndrome case with endometrial cancer is likely to be higher if the family’s mutation is in *MLH1 *or *MSH2 *than for *MSH6 *or *PMS2*; however, the limited information available to date suggests that families with *MLH1 *or *MSH2 *mutations may be in the minority of those identified through screening endometrial cancer cases. It is clear that genetic counselling needs to be sex-, age- and mutation-specific to help mutation-positive relatives understand their risk.

Some studies reported since publication of the EGAPP analysis have added to the evidence regarding clinical utility to the relatives of colorectal cancer patients diagnosed with Lynch syndrome. Two studies have found that screening may be cost-effective but the different mutation spectrum and risk profiles relevant to screening women with endometrial cancer mandate specific health-economic analysis for this scenario.^113^^,^ 114

Other evidence regarding screening of colorectal cancer patients is likely to be applicable to screening endometrial cancer patients. Some commentators have suggested that there is insufficient evidence that encouraging findings from research studies would be replicated if screening were to be rolled out to much larger numbers of patients and centres,^115^^,^ 116 and that advocates of screening have not taken sufficient account of patient preferences, psychosocial harms or inequity of access.^117^^,^ 118 A recent systematic review found that uptake of genetic testing among first degree relatives of people with Lynch syndrome may be only 34%-52%;^119^ this level may be too low to meet criteria for cost-effectiveness of screening programmes.^113^ Recent studies of US cancer centres that have taken up reflex testing of all newly diagnosed colorectal cancer patients have found evidence of heterogeneity in approach, difficulties in ensuring adherence to the screening and follow-up protocol by all the clinical teams involved, and concerns about patients’ willingness to comply with follow-up recommendations and/or insurers’ willingness to pay for testing.^25^^,^ 120 ^,^ 121 Factors important for a successful programme include good communication within the multidisciplinary team, early involvement and integration of the genetics team, and clear protocols and responsibilities for contacting and following up patients and their at-risk relatives.^121^^,^ 122

## Gaps in current evidence

An important gap in the evidence base is the relative paucity of studies on unselected endometrial cancer patients not restricted by age. The total number of mutation carriers identified in such studies to date is small, and only one of the two prospective studies was able to carry out mutation testing in almost all those with suggestive tumour test results, so it is difficult to estimate the level of confidence in findings such as mutation spectrum and age of onset.^2^^,^ 3 ^,^ 4 ^,^ 19 ^,^ 82 ^,^ 83 There is very little data on non-white population groups.

There are also uncertainties about the performance of IHC and MSI in analysis of endometrial tumours, as most published data relate to colorectal tumours. There is a need for specification of optimal test parameters, and reliable estimates of clinical sensitivity and specificity of these tests in unselected patients. Additional evidence is also needed to guide the choice of MSI and/or IHC in tumour testing of endometrial cancers. Cost considerations almost certainly rule out use of both tests in population screening programmes and some advocate the use of IHC alone,^13^ but the available information on proficiency testing for IHC suggests some cause for concern about quality (67 ^,^ 74 and www.nordiqc.org). Evaluation is also needed of the clinical validity and cost-effectiveness of criteria (such as methylation testing, or IHC results combined with features such as age at onset and family history) to exclude some women with abnormal tumour test results from germline DNA testing.

For germline DNA testing, optimum protocols for mutation detection should be determined, especially for the *PMS2 *gene. As massively parallel next-generation sequencing technologies begin to enter the clinical arena, the potential implications for the cost (and therefore availability) of clinical DNA sequencing will also need to be taken into account.

If screening programmes for newly diagnosed endometrial cancer patients become more widely implemented, further evidence is needed on the acceptability of tumour and germline DNA testing to these women, the likelihood that those who test positive will comply with advice on risk reduction, and the counselling of individuals with suggestive tumour testing results, but in whom no Lynch syndrome mutation is found. Recent evidence suggests that the risks associated with MMR mutation-negative “Lynch-like syndrome” may be intermediate between average population risks and risks in those with confirmed Lynch syndrome.^123^

Finally, there is a need for health-economic analysis that, as well as using appropriate assumptions (as discussed above), takes into account the costs and benefits of genetic counselling, mutation testing, surveillance and preventive interventions in the relatives of the index case.
